# Unmasking *Candida viswanathii* in Panel-Negative Candidemia Through Integrated MALDI-TOF MS and FTIR Spectroscopy

**DOI:** 10.3390/pathogens15070724

**Published:** 2026-07-09

**Authors:** Elena De Carolis, Terenzio Cosio, Carlotta Magrì, Marialaura Del Mondo, Riccardo Torelli, Flora Marzia Liotti, Paola Bernaschi, Tiziana D’ Inzeo, Giovanni Vento, Maurizio Sanguinetti

**Affiliations:** 1 Dipartimento di Scienze di Laboratorio ed Ematologiche, Fondazione Policlinico Universitario A. Gemelli IRCCS, 00168 Rome, Italy; terenziocosio@gmail.com (T.C.); carlottamagri97@gmail.com (C.M.); marialaura.delmondo@policlinicogemelli.it (M.D.M.); riccardo.torelli@policlinicogemelli.it (R.T.); tiziana.dinzeo@unicatt.it (T.D.I.); maurizio.sanguinetti@unicatt.it (M.S.); 2 Microbiology and Diagnostic Immunology Unit, Bambino Gesù Children’s Hospital, IRCCS, 00165 Rome, Italy; paola.bernaschi@opbg.net; 3 Neonatology Unit, Department of Woman and Child Health and Public Health, Fondazione Policlinico Universitario ‘Agostino Gemelli’ IRCCS, 00168 Rome, Italy; giovanni.vento@policlinicogemelli.it

**Keywords:** *Candida viswanathii*, candidemia, rare fungal infection, Fourier transform infrared spectroscopy, MALDI-TOF MS

## Abstract

Background: Rare fungal infections may represent under-recognized causes of healthcare-associated sepsis, particularly when caused by emerging or difficult-to-identify pathogens. We aimed to characterize *Candida viswanathii* isolates recovered in the setting of panel-negative candidemia and to assess the contribution of an integrated diagnostic workflow. Methods: We investigated seven *C. viswanathii* isolates overall, including three recovered at our institution from blood, urine, and bronchoalveolar lavage of a NICU patient, as well as four bloodstream isolates from a second pediatric center included for comparison. Isolates were analyzed by culture and microscopy, three MALDI-TOF MS platforms, internal transcribed spacer sequencing, Fourier transform infrared (FTIR) spectroscopy and antifungal susceptibility testing. Results: *C. viswanathii* was repeatedly recovered from blood, urine and bronchoalveolar lavage, while the FilmArray BCID2 panel remained negative. All MALDI-TOF MS systems with updated databases correctly identified the yeast at the species level; identification was confirmed by sequencing. Fourier transform infrared analysis showed clustering of clinical isolates and clearly separated *C. viswanathii* from related *Candida* species. All isolates exhibited low MICs to echinocandins and amphotericin B as well as moderately elevated fluconazole MICs (2–4 mg/L). Conclusion: This study supports the use of explicit diagnostic algorithms for rare fungal pathogens in yeast-positive, syndromic panel-negative blood cultures. In this setting, updated MALDI-TOF MS libraries and FTIR spectroscopy may provide useful adjunctive support for the recognition and phenotypic discrimination of atypical yeasts within an integrated laboratory workflow.

## 1. Introduction

Over the last decade, clinical microbiology has increasingly adopted “fast microbiology” pathways, including rapid syndromic molecular panels, chromogenic media as early screening, and automated phenotypic/biochemical identification, designed for speed and for the most prevalent pathogens [1,2]. This optimization improves turnaround time (TAT) for common pathogens, but it creates a predictable diagnostic blind spot for rare or cryptic fungi. Syndromic multiplex testing performed directly on positive blood culture provides rapid identification of most common bacterial bloodstream pathogens, thereby enabling earlier antimicrobial optimization [3,4]. Despite that, syndromic blood-culture panels are necessarily limited to a predefined target list; a yeast can be present in culture yet remain “panel-negative” simply because it is not included, and this may lead to premature closure if a negative panel is misinterpreted as excluding clinically relevant fungi [5]. Second, conventional phenotypic and biochemical systems often collapse uncommon species into common congeners because decision rules and reference datasets are tuned to high-prevalence taxa, not to rare species with overlapping phenotypes [6,7,8,9]. Third, chromogenic media can provide supportive clues but may not reliably discriminate closely related species across different formulations and reading conditions, so chromogenic appearance should not be used as a stand-alone identification criterion [10,11]. These limitations mean that “fast” does not always translate into “correct,” especially for yeasts where accurate species assignment is epidemiologically and therapeutically consequential. *Candida viswanathii* exemplifies this rare-but-relevant diagnostic gap.

*Candida viswanathii* was first described in 1959 from a case of meningitis in India and, again, in the 1970s from a second cerebrospinal fluid (CSF) isolate in the same country [12,13]. For several decades, it remained little more than a taxonomic curiosity, defined by morphology and biochemical features, with only sporadic clinical reports [12,13,14,15,16,17,18]. More recent series, however, indicate that it is increasingly recognized due to the improvement in diagnostic methods, particularly with the incorporation of molecular sequencing and updated matrix-assisted laser desorption/ionization time-of-flight (MALDI-TOF) mass spectrometry (MS) databases [14,15,16,17,18]. Phylogenetically close to *Candida tropicalis* (*C. tropicalis*), *C. viswanathii* appears to occupy environmental niches across South and Southeast Asia yet is easily overlooked by workflows, optimized for the most prevalent *Candida* species [19]. Whole-genome comparison demonstrated a clear separation between the two species. The estimated genome size differed markedly, being approximately 14.6 Mb for *C. tropicalis* and 24.2 Mb for *C. viswanathii*, with the latter showing an approximately 1.7-fold larger genome. Consistently, fast Average Nucleotide Identity (ANI) analysis yielded an ANI value of 78.12%, with only 38% of the genome alignable between the two species [18]. From a practical diagnostic perspective, the distinction between *Candida viswanathii* and *C. tropicalis* remains difficult within routine phenotypic workflows. First, *C. viswanathii* can produce pseudohyphae and ovoid blastoconidia, including arrangements in chains. *C. tropicalis* also produces abundant pseudohyphae and blastoconidia along filamentous structures, and it may form true hyphae under suitable conditions. These patterns overlap substantially in routine microscopy. Their microscopic features are insufficiently discriminatory for reliable species-level identification [13,18]. In the largest early clinical series [15], conventional methods as VITEK 2 or API 20C AUX failed to differentiate the two species reliably; *C. viswanathii* assimilated sucrose and cellobiose but did not assimilate trehalose or raffinose, whereas *C. tropicalis* showed a variable assimilation pattern for these substrates, limiting the stand-alone value of biochemical testing for species-level discrimination [15]. Older phenotypic databases may also return to *C. viswanathii* under the historical name *Candida lodderae*, further complicating interpretation [20]. More broadly, commercial biochemical systems perform substantially less well for rare yeasts than for the commonest *Candida* species, which makes uncommon taxa particularly vulnerable to low-confidence or misleading phenotypic assignments [21]. Reliable identification typically requires sequencing of the internal transcribed spacer (ITS) or D1–D2 rDNA regions, or updated MALDI-TOF MS databases and other high-resolution typing methods as whole-genome sequencing (WGS) [14,15,16,17,18]. In particular, MALDI-TOF MS identification is nowadays a fundamental application in clinical microbiology laboratories. The method is based on the mass spectra microbial fingerprint, unique for each species, where the spectrum matched against a reference database allows the identification at the species level of a wide range of clinical microbial pathogens. Being introduced some decades ago is now widely used for secure, low-cost and fast identification from agar-grown bacteria and fungi as well as from biological samples (positive blood cultures) [22].

In recent years, FTIR spectroscopy has gained impact in real-time outbreak investigation both for bacteria and yeasts [23]. The method allows us to obtain a unique microbial fingerprint by infrared light absorption bands produced on the basis of microbial cell chemical bond vibrations. FTIR spectroscopy can differentiate between species and subspecies and is reported to be very appreciated for related pathogenic yeasts isolates clustering [24]. Interestingly, in 2025, the first European outbreak of *C. viswanathii* bloodstream infection was reported in a pediatric hospital in Italy, where fifteen isolates were recovered from blood cultures of consecutive neonatal intensive care unit (NICU) patients over a four-month period [18].

We describe an integrated laboratory approach combining MALDI-TOF MS identification, ITS sequencing, FTIR as an innovative typing method, and antifungal susceptibility testing, and we assess its utility for recognizing and characterizing *Candida viswanathii* during the first European candidemia outbreak caused by this species.

## 2. Materials and Methods

A total of seven *Candida viswanathii* isolates were included in the study. Four isolates were recovered in October 2025 from blood (FPG 2277/25, FPG 2289/25), urine (FPG 2296/25), and bronchoalveolar lavage cultures (FPG 2298/25), from a pediatric patient admitted to the neonatal intensive care unit (NICU) at Fondazione Policlinico Universitario A. Gemelli IRCCS (Rome, Italy). In addition, three bloodstream isolates from Bambino Gesù Children’s Hospital, IRCCS (Rome, Italy), namely OPBG 1, OPBG 2, and OPBG 3, were included as comparator isolates to support the microbiological investigation and the contextual interpretation of relatedness; these isolates had been previously characterized by WGS in the outbreak study by Vrenna et al. [18].

### 2.1. Microbiological Culture and Microscopy

With clinical suspicion of bloodstream infection (BSI) in a NICU patient, peripheral and central venous blood samples were collected and inoculated into paired aerobic and anaerobic blood culture bottles into the BACT/ALERT^®^ VIRTUO^®^ instrument (bioMérieux, Marcy l’Étoile, France). After seven hours of incubation, one aerobic bottle flagged positive, Gram staining was performed and in parallel it was subcultured on bromocresol green yeast agar (BCG) (Kyma, Padua, Italy), Chocolate Blood Agar (PVX), Tryptic Soy Agar (TSA) and MacConkey agar (bioMérieux, Marcy-l’Étoile, France) plates, as part of the routine work-up of positive blood cultures, in order to facilitate yeast recovery and preliminary morphological assessment while also excluding concomitant bacterial growth. All media were incubated at 35 ± 2 °C. Colony growth was assessed after 24–48 h.

### 2.2. MALDI-TOF MS Identification

In order to confirm the identification at the species level, MALDI-TOF mass spectrometry identification was performed using three platforms available at Policlinico Gemelli Hospital, Rome: the MALDI Biotyper^®^ (Bruker Daltonics, Bremen, Germany), the VITEK^®^ MS PRIME (bioMérieux, Marcy-l’Étoile, France), and the Autof MS2600 (Autobio Diagnostics, Zhengzhou, China). For the Bruker system, the direct transfer method was applied: a small portion of a fresh colony was spotted on a steel target, treated with 1 µL formic acid and 1 µL α-cyano-4-hydroxycinnamic acid (HCCA) matrix, and analyzed with MBT Compass Explorer 4.1 using the Bruker database (database version 4.1). Calibration was performed by the Bruker Bacterial Test Standard (BTS), and identification scores were interpreted as ≥2.0 (species level), 1.7–1.99 (genus level), or <1.7 (unreliable). For the VITEK^®^ MS PRIME, colonies were transferred onto a MS-DS target plate using a PICKME™ PEN, overlaid with 70% formic acid and HCCA matrix and analyzed with VITEK^®^ MS Software (v1.1.0–203571250) and IVD Database 3.2. *Escherichia coli* (*E. coli*) ATCC 8739 served for calibration, and confidence values ≥ 60% were considered species-level. For the Autof MS2600, spectra were acquired with Autof Acquirer Software (v2.0.196) and matched against the Autobio Library (V1120). Calibration was performed with the Autof MS Calibrator (ribonuclease, myoglobin, *E. coli* extracts). Identification scores ≥ 9.0 were species-level, 6.0–8.99 genus-level, and <7.0 unreliable.

### 2.3. Molecular Confirmation

Species identity was confirmed by sequencing the ribosomal DNA internal transcribed spacer (ITS1-5.8S-ITS2) region. DNA was extracted from fresh pure colonies using the ZymoBIOMICS™ DNA Miniprep Kit (Zymo Research, Irvine, CA, USA) according to the manufacturer’s instructions. Briefly, colonies were processed in ZR BashingBead™ Lysis Tubes containing ZymoBIOMICS™ Lysis Solution, mechanically disrupted, and centrifuged, and the recovered supernatant was filtered and purified on a Zymo-Spin™ IICR column. After sequential washing steps, DNA was eluted in DNase/RNase-free water and used for ITS PCR amplification and sequencing. PCR amplification was carried out with universal fungal primers ITS1 (5′-TCCGTAGGTGAACCTGCGG-3′) and ITS4 (5′-TCCTCCGCTTATTGATATGC-3′ [25]. Cycling conditions were as follows: initial denaturation at 95 °C for 3 min, followed by 35 cycles of 95 °C for 30 s, 55 °C for 30 s, and 72 °C for 1 min, with a final extension at 72 °C for 5 min using an Eppendorf Mastercycler nexus SX1 (Eppendorf, Hamburg, Germany). Amplicons were purified using the MiniElute PCR Purification Kit (Qiagen, Hilden, Germany) and sequenced with the BigDye Terminator v3.1 kit (Applied Biosystems, Foster City, CA, USA) on a SeqStudio™ Genetic Analyzer. Consensus sequences were analyzed using Chromas v2.6.6 and compared with reference sequences in GenBank using BLASTn (https://blast.ncbi.nlm.nih.gov/Blast.cgi) (accessed on 6 July 2026). ITS sequences from the four isolates were subsequently aligned with two *C. viswanathii* reference *strains* and three *C. tropicalis* sequences retrieved from GenBank using the ClustalW algorithm using MEGA software (version 10.2.6). Phylogenetic analysis was performed using the Neighbor-Joining method based on Maximum Composite Likelihood [26,27,28,29].

### 2.4. Fourier Transform Infrared Spectroscopy Analysis

Fourier transform infrared spectroscopy (FTIR) analysis was performed using the IR Biotyper^®^ system (Bruker Daltonics, GmbH, Bremen, Germany). Seven different clinical isolates of *Candida viswanathii* (three from different clinical sources; blood, urine and BAL fluid of the same patient and four from blood cultures of patients from a different Italian hospital implicated in the same outbreak as control) and identified by whole-genome sequencing (WGS) as *C. viswanathii* were included in the analysis [18]. Moreover, *Candida auris* (*C. auris*) clades I–IV, *Candida parapsilosis* (*C. parapsilosis*) and *C. tropicalis* isolates belonging to the Mycology lab collection at Fondazione Policlinico Gemelli, Rome, were included in the analysis. All these reference control strains had undergone whole-genome sequencing, providing species-level confirmation before inclusion in the FTIR analysis. Isolates were grown on Sabouraud Dextrose agar (SDA) (Kyma, Padua, Italy) plates for 24 h at 37 °C, and sample preparation was carried out following the manufacturer’s guidelines. Briefly, yeast cells were suspended in 70% ethanol and homogenized by vortexing twice before and after the addition of 50 µL of deionized water in 1.5 mL microcentrifuge tubes containing metal beads. For technical replicates, 15 µL of the homogenized suspension was spotted in quintuplicate onto a silicon sample plate and air-dried before analysis. To assess inter-run variability, three independent biological replicates were performed. Each analytical run included two infrared test standards in duplicate as quality controls. Spectra were acquired in the carbohydrate-typing region (1300–800 cm^−1^) using the IR Biotyper^®^ software with default acquisition and preprocessing parameters, following established quality control criteria. Spectral reproducibility among replicates was verified based on label coherence and cluster purity metrics. For each isolate meeting quality standards, an average spectrum was generated from replicate spectra using OPUS version 8.2.28 software (Bruker Daltonics). The resulting spectral data were examined by principal component analysis (PCA) and linear discriminant analysis (LDA) to investigate clustering patterns and inter-species relationships. Hierarchical cluster analysis (HCA), based on the correlation average linkage method, was applied to evaluate inter-isolate similarity and establish relatedness among samples. Spectral profiles of the clinical isolate were compared with selected FTIR reference spectra from sequence-validated *C. tropicalis* (CT-35/196/128/48), *C. auris* (clades I–IV: B11103, B11220, B11221, B11244), and *C. parapsilosis* (including azole-resistance mutants 16R Y132F, CP-AR-365 Y132F-R398I, 10S R398I, 20S WT) strains [30,31,32].

### 2.5. Retrospective Analysis of 2025 Candida tropicalis Isolates and Infecion Control Interventions

Since the index isolate was identified as *Candida viswanathii* by all three MALDI-TOF MS platforms used in our laboratory, albeit with different confidence scores, we retrospectively reviewed blood culture isolates reported as *C. tropicalis* from March 2025 to September 2025. This review was performed after local updating of the MALDI-TOF MS libraries, which had been expanded using the reference spectral profiles generated from the seven *C. viswanathii* isolates included in the present study. The aim was to assess whether earlier bloodstream isolates originally classified as *C. tropicalis* might in fact have represented unrecognized *C. viswanathii* before this case prompted targeted reassessment. Moreover, as measures control, blood cultures were performed in the subsequent days in the NICU in order to verify other possible cases related to the present one. Environmental swabs (monitors, NICU incubators, linens) were added as further infection control measures.

### 2.6. Antifungal Susceptibility Testing

Antifungal susceptibility was assessed on all the *C. viswanathii* isolates using the Sensititre YeastOne^®^ ITAMYUCC custom panel (Thermo Fisher Scientific, Oakwood, GA, USA), according to the Clinical and Laboratory Standards Institute (CLSI) broth microdilution method [33]. Yeast inocula were prepared in sterile saline and adjusted to 0.5 McFarland, then diluted in RPMI 1640 medium, following the manufacturer’s protocol. Panels were incubated at 35 ± 2 °C for 24 h, until there was visible growth in the control wells. Minimum inhibitory concentrations (MICs) were determined as the lowest antifungal concentration that prevented a color change from blue (no growth) to pink/red (growth) and by visual evaluation of growth sign in wells. Since no species-specific breakpoints exist for *C. viswanathii*, MICs were reported descriptively.

## 3. Results

### 3.1. Blood Culture Isolation and Microscopic Examination of Candida viswanathii

**Figure 1:**
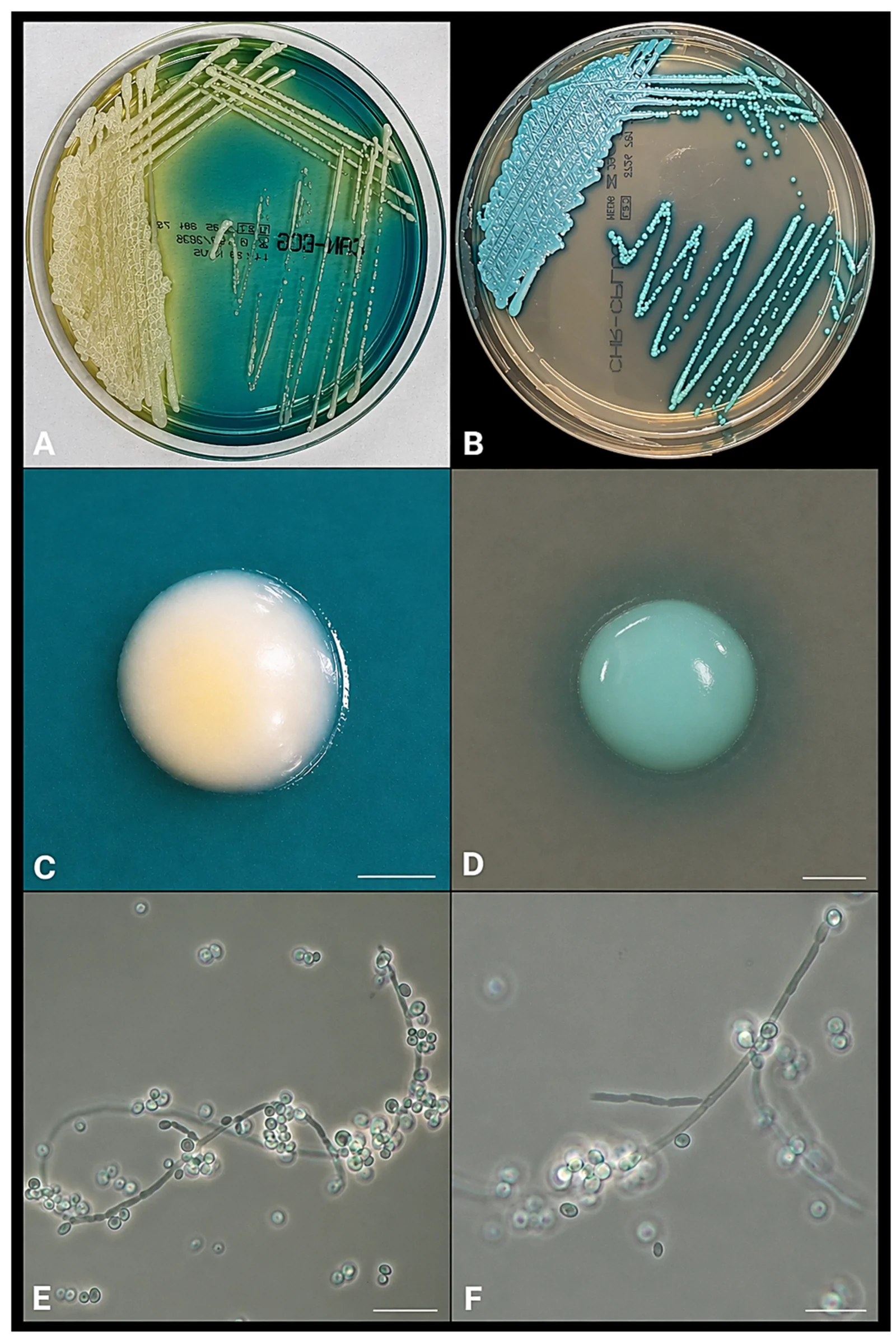
Morphology of *Candida viswanathii*. (**A**) On CAN–BCG (37 °C, 48 h), the isolate formed cream-white yeast colonies. (**B**) On CHROMagar™ Candida, colonies were smooth and pale bluish turquoise. (**C**,**D**) Single-colony focus of *C. viswanathii* on BCG and CHROMagar™ Candida scale bars, 3 mm. (**E**,**F**) Phase-contrast microscopy at 40× magnification showed oval budding yeast cells (3–6 µm), with pseudohyphal elements (2–4 µm) displaying septal constrictions; panel (**E**) shows abundant blastoconidia along short pseudohyphae, whereas panel (**F**) shows elongated pseudohyphae with sparse blastoconidia. Chlamydospores were not observed. Scale bars, 20 µm.

After microscopic examination of a budding yeast, the BIOFIRE^®^ Blood Culture Identification 2 (BCID2) Panel (FilmArray^®^) tested negative. After 16 h of incubation, smooth, cream-colored colonies were visible on CAN-BCG, while on TSA and PVX, colonies showed creamy-white pigmentation and radial margins forming a star-like outline. No bacterial contaminants were observed on parallel TSA or MacConkey plates. The isolate was provisionally classified as a *Candida* species based on morphology and subsequently subjected to mass spectrometry identification by MALDI-TOF MS on the same day as *Candida viswanathii* and molecular confirmation via ITS rDNA sequencing. Macroscopic examination revealed confluent, dense, and rugose/cerebriform cream-white yeast colonies in the primary streak, and along secondary streaks discrete, opaque, matte-dry colonies that slightly spread along the loop tracks; a yellow halo encircled the heaviest growth against the teal-green medium, consistent with local acidification of the indicator dye (Figure 1A). Subculture on CHROMagar™ Candida were performed and evaluated at 48 h, demonstrating smooth and pale bluish turquoise colonies (Figure 1B). Moreover, in side-by-side plating experiment, the reference *Candida tropicalis* ATCC 750 strain produced colonies with a chromogenic appearance comparable to that observed for the study isolate, did not allow a reliable visual distinction between *C. viswanathii* and *C. tropicalis* based on chromogenic medium (Figure S1). Microscopic examination of lactophenol blue smears revealed oval budding yeast cells (3–6 µm) together with pseudohyphal elements (2–4 µm wide) showing constrictions at the septa, as well as blastoconidia arranged singly and in small clusters along short pseudohyphal segments (Figure 1C,D). Chlamydospores were not observed in these preparations.

### 3.2. MALDI-TOF MS-Based Identification of Candida viswanathii

All clinical isolates were identified as *Candida viswanathii* by the three MALDI-TOF MS platforms, although the accuracy of identification differed markedly between systems and across replicate spectra. VITEK^®^ MS PRIME provided consistent species-level identification with 99.9% confidence for all tested isolates as *C. viswanathii*.

With the Bruker Biotyper^®^, one run yielded a high-confidence *C. viswanathii* identification (score 2.03; *C. tropicalis* was the closest alternative match at substantially lower score), in agreement with sequencing. However, additional Bruker acquisitions showed greater variability, showing limited score separation between *C. viswanathii* and *C. tropicalis*; in one spectrum, the top hit list was dominated by *C. tropicalis* (best score 1.7), highlighting the proximity of these taxa in the reference library and the risk of low-confidence assignments when spectra quality or database representation is suboptimal.

On the Autof MS2600 (Autobio), *C. viswanathii* was consistently ranked as the first match, but identification scores were heterogeneous (6.0–9.2 across replicate spots). Across low-score Autof spectra, *C. tropicalis* recurrently appeared among the closest alternative matches. Taken together, these findings indicate that identification at the species level for *C. viswanathii* can be correctly assigned by MALDI-TOF MS, but confidence may vary by platform and spectrum, with *C. tropicalis* representing the most frequent near-neighbor; therefore, final species confirmation in this cluster relied on concordant MALDI-TOF calls supported by ITS sequencing or on updated MALDI-TOF databases. Raw data are included in Figure S2.

### 3.3. Molecular Identification of Candida viswanathii

**Figure 2:**
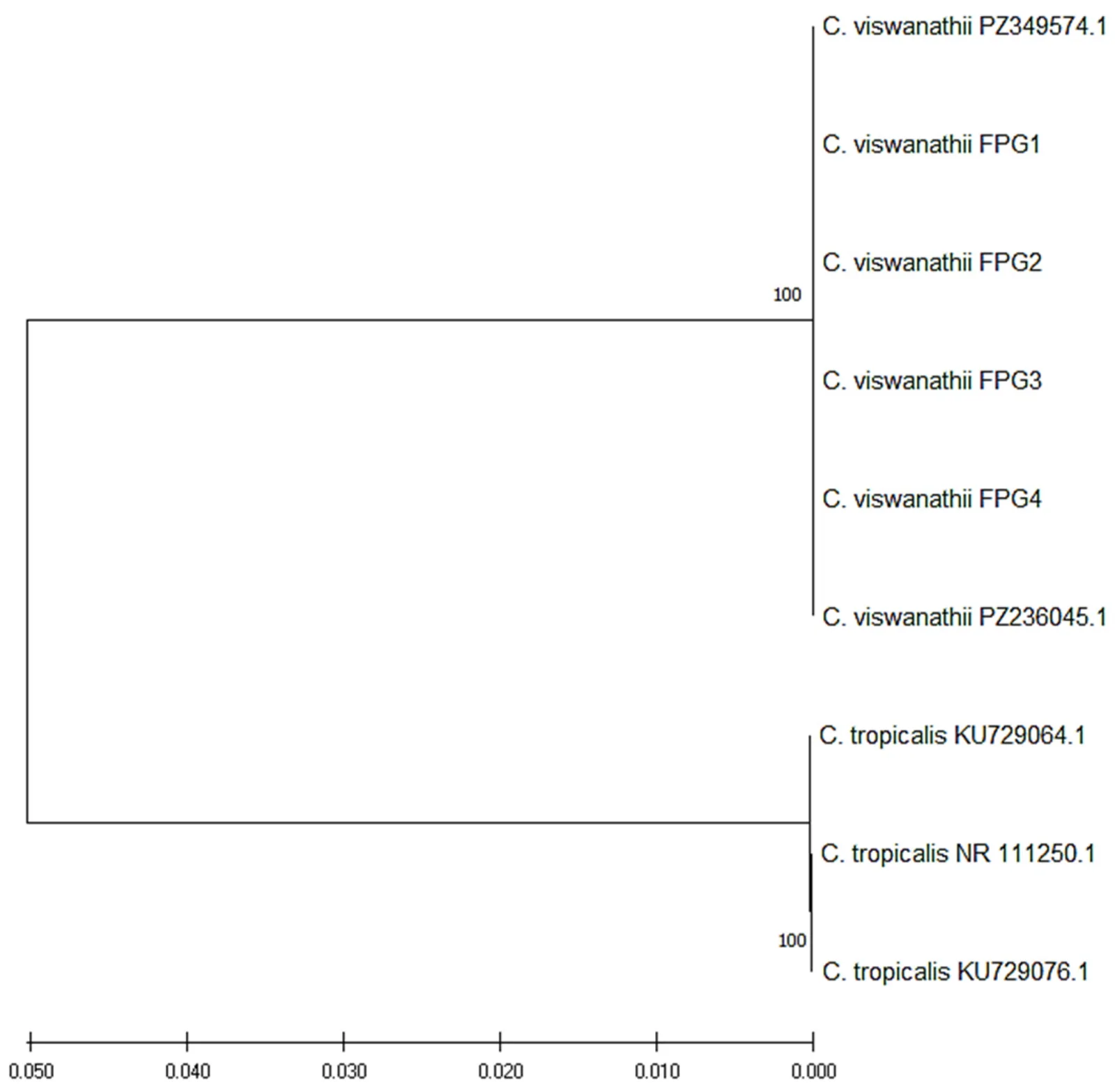
Neighbor-Joining phylogenetic tree based on partial ITS rDNA sequences including the four *C. viswanathii* isolates obtained in this study (FPG1–FPG4), two reference *C. viswanathii* and *C. tropicalis* sequences retrieved from GenBank. Bootstrap values are shown at the nodes.

The consensus ITS sequence was matched against the NCBI BLASTn algorithm (https://blast.ncbi.nlm.nih.gov/Blast.cgi, accessed on 2 October 2025), showing 100% identity with *Candida viswanathii* reference strain CBS 7889 (GenBank accession number KY102513.1). The dendrogram (Figure 2) showed a clear separation between the two *Candida* species that are grouped in different branches of the tree.

### 3.4. Fourier Transform Infrared Spectroscopy Analysis (FTIR)

**Figure 3:**
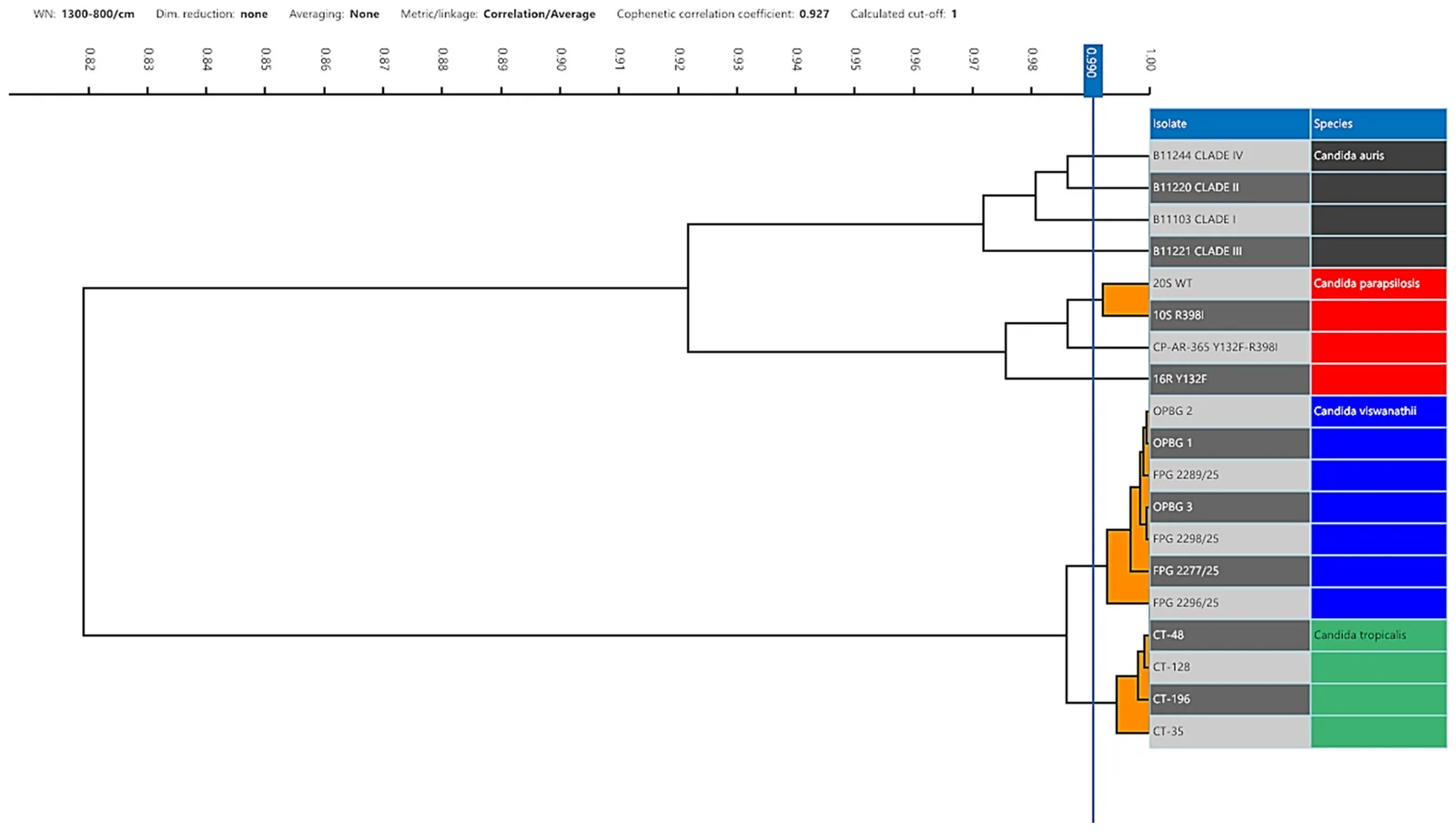
FTIR dendrogram showing hierarchical clustering of *Candida* isolates, including the clinical *C. viswanathii*, based on spectral similarity (1300–800 cm^−1^ range). Specific isolate identifiers are shown in the adjacent “Isolate” column, reported with different species-specific color, and including the *C. viswanathii* study isolates FPG 2277/25, FPG 2289/25, FPG 2296/25, FPG 2298/25, and OPBG 1–3. Distances were calculated using Euclidean metrics and average linkage. Cut-off value (0.990) was calculated based on the twelve reference control strains included in the analysis.

**Figure 4:**
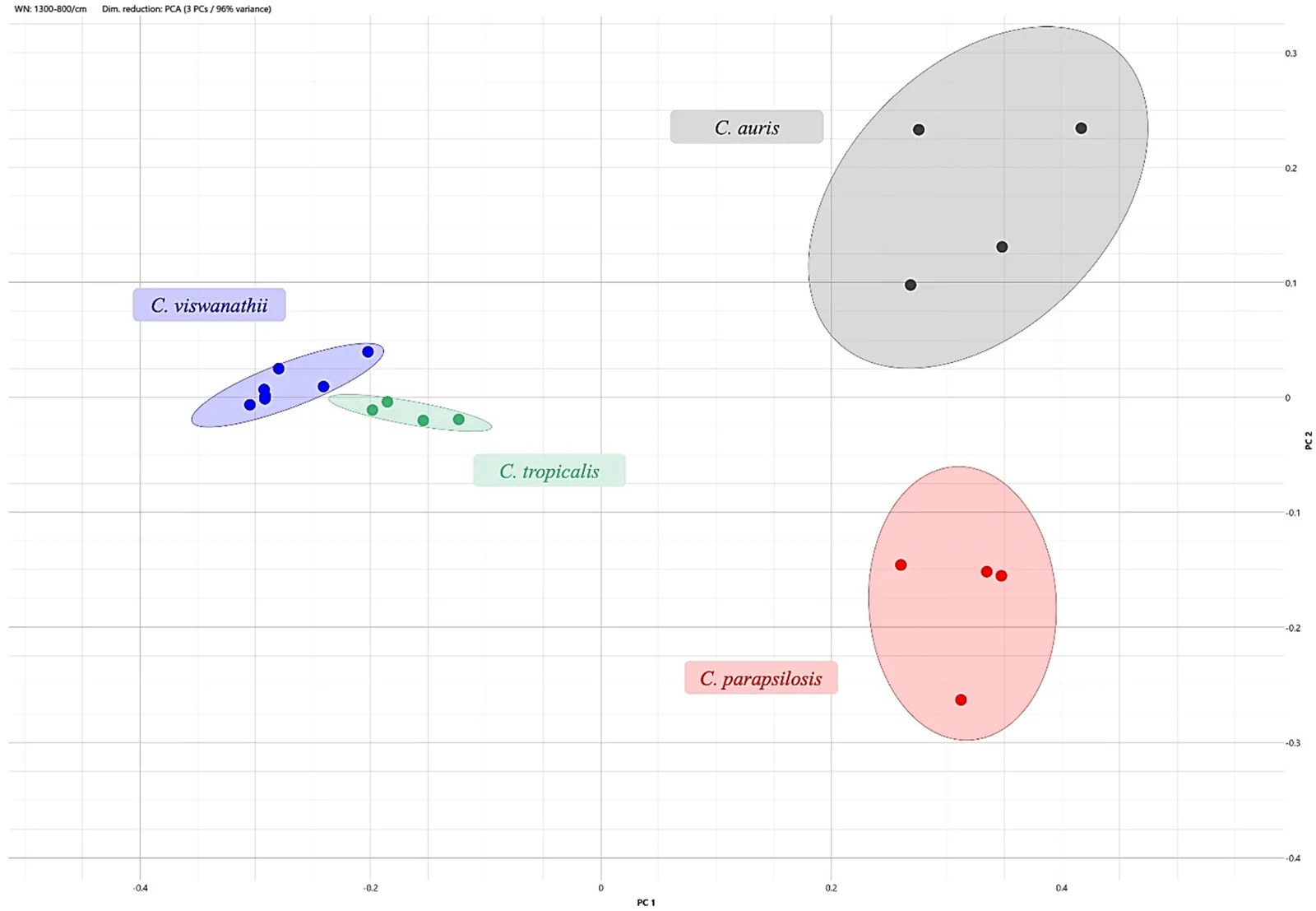
Principal component analysis (PCA) plot of FTIR spectra acquired in the 1300–800 cm^−1^ carbohydrate-region window. Each dot represents one isolate projected into the PC1/PC2 plot according to its FTIR spectral profile. Colors indicate *Candida viswanathii* in blue, *Candida tropicalis* in green, *Candida parapsilosis* in red and *Candida auris* in gray. The ellipses correspond to the software-generated 95% confidence ellipses for each species group and summarize group dispersion in the displayed PCA plot. PC1 and PC2 together accounted for 96% of the total spectral variance.

**Figure 5:**
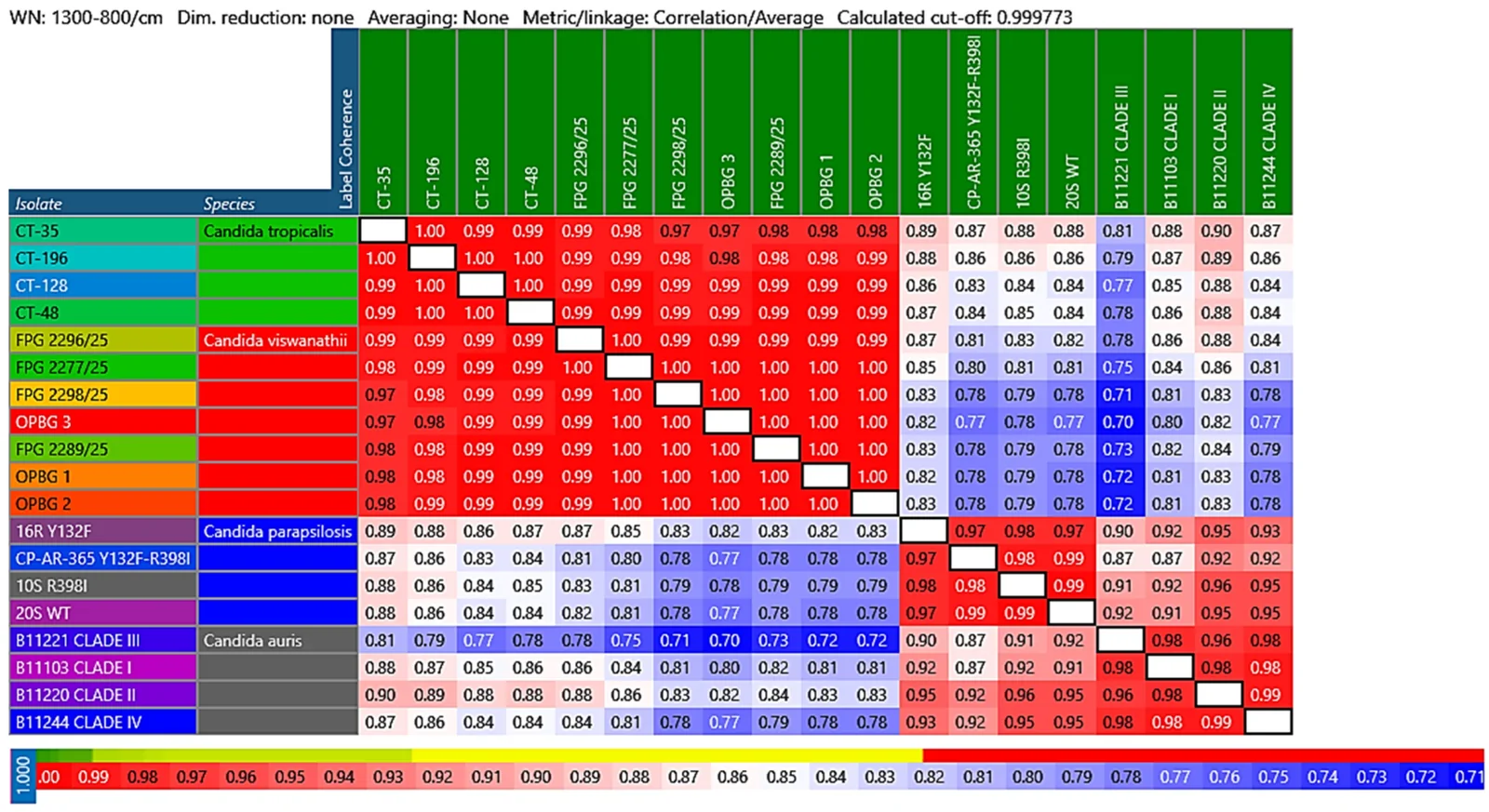
FTIR spectral similarity of *Candida* isolates (1300–800 cm^−1^). Pairwise similarities were calculated using correlation as the similarity metric, with average linkage hierarchical clustering. Rows and columns contain the same isolates, and the diagonal corresponds to self-comparisons. The software-calculated cut-off was 0.999773. The color scale at the bottom represents pairwise spectral similarity, ranging from 0.71 in blue, indicating lower similarity, to 1.00 in red, indicating higher similarity. The numbers displayed within the matrix are rounded to two decimal places for visualization. The matrix shows species-associated blocks corresponding to *Candida tropicalis* isolates CT-35, CT-196, CT-128 and CT-48; *Candida viswanathii* isolates FPG 2296/25, FPG 2277/25, FPG 2298/25, OPBG 3, FPG 2289/25, OPBG 1 and OPBG 2; *Candida parapsilosis* comparator isolates 16R Y132F, CP-AR-365 Y132F-R398I, 10S R398I and 20S WT; and *Candida auris* clade I–IV isolates B11103, B11220, B11221 and B11244. The *C. viswanathii* isolates, including the clinical isolates from our center and those from the outbreak investigated at another Italian center, formed a coherent block, with displayed intra-group similarity values of 0.99–1.00. The “Local Coherence” bar (from dark green to red color) is a software-generated annotation in the matrix and was not used as an independent taxonomic or analytical cut-off.

FTIR analysis enabled high-resolution differentiation between the clinical *Candida viswanathii* isolate and closely related *Candida* species. Hierarchical cluster analysis by correlation average linkage revealed distinct groups for the different *Candida* species (Figure 3). The clinical isolates assigned to *Candida viswanathii* grouped with the *C. viswanathii isolates* from the outbreak investigated at the other hospital, which had been processed and characterized by WGS. In the FTIR analysis, these isolates defined a coherent *C. viswanathii*-associated spectral cluster within the analyzed dataset. This cluster was separated from those corresponding to *C. tropicalis*, *C. parapsilosis* and *C. auris*, although *C. tropicalis* showed the closest spectral relationship to the *C. viswanathii* group among the comparator species. PCA showed that the clinical *C. viswanathii* isolates grouped together with the WGS-characterized outbreak isolates in the PCA plot, supporting their separation from the other species included in the analysis (Figure 4). Moreover, the FTIR similarity matrix generated from the 1300–800 cm^−1^ carbohydrate-region spectra showed that the clinical *Candida viswanathii* isolates grouped with the WGS-characterized outbreak isolates from the other hospital. Within the *C. viswanathii* group, displayed correlation values were 0.99–1.00. *C. tropicalis* formed the closest neighboring spectral group, with cross-group similarities to *C. viswanathii* mainly displayed as 0.97–0.99. However, clustering was performed by the software using full-precision correlation values, with a calculated dataset-specific cut-off of 0.999773. *C. parapsilosis* and *C. auris* showed lower similarity to *C. viswanathii* in the displayed matrix, with rounded values not exceeding 0.90. This analysis supported the FTIR-based grouping of the clinical *C. viswanathii* isolates with the WGS-characterized outbreak isolates and confirmed that *C. tropicalis* was the closest comparator species in the dataset (Figure 5). Together, this analysis shows that *C. viswanathii* and *C. tropicalis*, two closely related yeast species within the *Candida/Lodderomyces* clade of the family Debaryomycetaceae [19,34], remain clearly distinguishable by FTIR biochemical fingerprinting. Raw data are reported in Figure S2.

### 3.5. Retrospective Re-Evaluation of Isolates Initially Identified as Candida tropicalis

In the period from March to September 2025, six bloodstream isolates previously identified as *Candida tropicalis* were retrospectively re-analyzed after updating the MALDI-TOF MS libraries of Bruker Biotyper^®^ and Autof MS2600 (Autobio) platforms with spectral data derived from the seven study isolates. Re-evaluation confirmed that all six isolates were *Candida tropicalis* and not *C. viswanathii*. As part of the infection control investigation, follow-up blood cultures obtained in the NICU over the subsequent days were negative, with no evidence of additional related cases. Environmental swabs from monitors, incubators, and linens were likewise negative, arguing against an identifiable environmental reservoir.

### 3.6. Antifungal Susceptibility Results of Candida viswanathii Strains

The MIC results on the seven different clinical isolates of *Candida viswanathii* revealed low values across all antifungal classes, except for fluconazole (Table 1). Echinocandins, including anidulafungin, micafungin, and caspofungin, showed the highest activity, with MICs ranging from 0.01 to 0.06 µg/mL for anidulafungin, ≤0.008 to 0.03 µg/mL for micafungin, and 0.06 to 0.12 µg/mL for caspofungin. The azole group, comprising isavuconazole, posaconazole, voriconazole, and itraconazole, displayed consistently low MICs, between 0.06 and 0.25 µg/mL for the first four agents, while fluconazole exhibited slightly higher values (2–4 µg/mL), though still within the expected susceptibility range. Amphotericin B also demonstrated low MICs, between ≤0.12 and 0.5 µg/mL across all specimens. Comparable susceptibility patterns were observed regardless of the sample type (blood, urine, or BAL), suggesting that all isolates share a homogeneous antifungal profile without evidence of resistance to any of the tested agents.

## 4. Discussion

### 4.1. Diagnostic Pitfalls in Rare Yeasts Identification

A yeast-positive blood culture with a negative syndromic panel represents an increasingly high-risk diagnostic junction. In this setting, accurate species assignment is essential for outbreak recognition and appropriate antifungal management, yet routine workflows may systematically underperform for rare taxa. Here, we integrate multi-platform MALDI-TOF MS, ITS sequencing, FTIR fingerprinting, and susceptibility testing to define both identification reliability and sample clustering for *Candida viswanathii*.

From a diagnostic standpoint, this case underscores how *C. viswanathii* still falls through the gaps of “fast microbiology” workflows. Phenotypic identification systems systematically collapse *C. viswanathii* into *C. tropicalis*, because their assimilation rulesets and databases are optimized for prevalent species within the *Candida/Lodderomyces* clade and lack calibrated profiles for rarer taxa [15,16]. In our case, the initial blood culture flagged as positive for yeasts, but the BIOFIRE^®^ Blood Culture Identification 2 (BCID2) Panel was negative, reflecting the absence of *C. viswanathii* from its target list. This scenario, where microscopy is positive for yeast while the molecular panel is negative, can lead to premature closure if clinicians associate a negative panel with “no relevant pathogen”. One common method used for discriminating against *Candida* spp. is chromogenic medium, despite presenting several limitations [10]. Interestingly, our chromogenic findings only partially aligned with those recently reported by Vrenna et al. [18], who observed a stable deep-blue colony color for *C. viswanathii* on chromID Candida that was distinguishable from the blue-green hue of *C. tropicalis* under their conditions. In our side-by-side plating, a reference *Candida tropicalis* ATCC 750 strain yielded colonies with an indistinguishable chromogenic appearance under routine laboratory conditions, indicating that, in our setting, CHROMagar™ Candida alone did not allow reliable visual separation of *C. viswanathii* from *C. tropicalis* (Figure S1). These discrepant observations suggest that the discriminatory performance of chromogenic media for *C. viswanathii* versus *C. tropicalis* is highly dependent on medium formulation, incubation temperature and reading conditions, and that chromogenic reactions should be interpreted as supportive clues rather than stand-alone identification criteria. In this scenario, MALDI-TOF MS provides an important bridge between conventional phenotypic methods and sequencing, but its performance is critically dependent on database curation. In earlier Indian reports, Bruker MALDI-TOF MS using the v3 database returned low-confidence *C. tropicalis* calls for *C. viswanathii* isolates until reference spectra from sequence-proven strains were added [15,16,17]. Moreover, Vrenna et al. [18] were unable to identify *C. viswanathii* by MALDI-TOF. By contrast, in our investigation, three independent MALDI-TOF MS systems, equipped with updated libraries that explicitly include *C. viswanathii*, concordantly identified the yeast. Moreover, after database implementation by additional *C. viswanathii* spectra to the libraries, routine identification became consistently high-confident across replicates, showing reliable identification scores for all the technical spot replicates, reducing the risk of low-confidence assignments in favor of *C. tropicalis*.

Published misidentifications further complicate the epidemiologic picture in the opposite direction: two nail isolates initially labeled “*C. viswanathii*” by PCR-fragment size polymorphism was reclassified as *C. tropicalis* and *C. orthopsilosis* by ITS rDNA sequencing, while Polymerase Chain Reaction-Restriction Fragment Length Polymorphism (PCR-RFLP) identified the first as *C. tropicalis* and left the second unidentified [35]. Together, these observations not only suggest that *C. viswanathii* is well within the reach of current MALDI-TOF technology when libraries are appropriately curated, but also that sequence-based methods remain essential for correct identification in absence of well-curated MALDI-TOF MS databases. In high-risk settings such as NICUs or outbreak investigations, a yeast that is BCID-negative yet repeatedly identified by MALDI-TOF MS as *C. viswanathii* or as a rare *Candida* species, especially in the presence of low-confidence scores, should suggest performing ITS and/or D1-D2 sequencing.

### 4.2. Added Value and Boundaries of FTIR Fingerprinting

Our study also highlights, for the first time, the potential of FTIR spectroscopy as an emerging adjunctive typing tool for common and rare yeasts. FTIR is gaining increasing traction in medical mycology as a rapid, high-throughput phenotypic fingerprinting technique, and in our setting, it reliably separated *C. viswanathii* from closely related species such as *C. tropicalis*. Moreover, our FTIR data complement those reported by Vrenna et al. [18], who used the same IR Biotyper platform to recognize a clonal *C. viswanathii* outbreak once the index isolated had been confirmed by WGS. In their setting, isolates formed a very tight FTIR cluster with minimal intra-cluster variability, consistent with a single outbreak lineage. Nevertheless, we focused on both species-level discrimination and source attribution; our *C. viswanathii* isolates grouped tightly with sequence-validated reference strains, while *C. tropicalis* isolates constituted the nearest cluster and remained clearly separated from *C. parapsilosis* and *C. auris*. The apparent proximity between *C. viswanathii* and *C. tropicalis* should be interpreted as proximity in the PCA plot of the carbohydrate-region FTIR data. The convergence of these independent datasets reinforces FTIR spectroscopy as a robust, rapid front-line tool to flag atypical yeasts and to support outbreak hypotheses, though results should be interpreted in conjunction with WGS data. At the same time, FTIR remains a phenotypic method dominated by cell-wall and membrane signatures, suited for species-level discrimination and rapid screening during suspected outbreaks, but it cannot prove clonality or exclude microevolution within a species [36,37]. In our view, FTIR should therefore be used as a rapid front-line discriminator to flag atypical or unexpected yeasts and to support the hypothesis of a common source, particularly in high-risk settings, but always in combination with sequencing and, where available, higher-resolution typing methods.

### 4.3. Susceptibility Profile and Clinical Implications

Regarding antifungal susceptibility, the emerging picture of *C. viswanathii* is one of generally preserved polyene and echinocandin activity with more variable azole susceptibility. The largest Indian series reported a temporal drift towards elevated fluconazole MICs over the course of an outbreak, whereas amphotericin B and echinocandins remained within low MIC ranges [15]. Our outbreak aligned with this broader pattern and with Vrenna et al. [18], where MICs to echinocandins were uniformly low and amphotericin B remained favorable; other triazoles had low MICs, while fluconazole clustered at moderately elevated values in the absence of species-specific breakpoints. Small differences between blood, urine and BAL isolates are most plausibly explained by broth microdilution variability and azole trailing rather than genuine resistance heterogeneity. Clinically, these data support current pediatric and neonatal recommendations that prioritize echinocandins or amphotericin B as first-line therapy for invasive candidiasis, reserving azoles for step-down in clinically stable patients with documented susceptibility [38,39,40]. However, the combination of misidentification as *C. tropicalis* and rising fluconazole MICs in some series raises the risk of inappropriate fluconazole monotherapy during step-down. In the absence of species-specific breakpoints or established ERG11/FKS-mediated resistance mechanisms, individualized MIC-based interpretation and targeted genomic surveillance are warranted, particularly when managing clusters or recurrent infections.

### 4.4. C. viswanathii vs. C. tropicalis

The distinction between *C. viswanathii* and *C. tropicalis* is not merely taxonomic, but has direct diagnostic, epidemiological, and potentially therapeutic implications. In our setting, classification of these isolates as *C. tropicalis* would probably have obscured the recognition of an unusual cluster, allowing the episode to be interpreted as candidemia caused by a common *Candida* species. First, phenotypic identification systems systematically collapse *C. viswanathii* into *C. tropicalis*, because their assimilation rulesets and databases are optimized for prevalent species within the *Candida/Lodderomyces* clade and lack calibrated profiles for rarer taxa [15,16]. Then, *C. viswanathii* can be misidentified as *C. tropicalis* by MALDI-TOF MS and undetected by the FilmArray BCID2 panel [18]. Moreover, *C. viswanathii* isolates showed fluconazole MICs of 2–4 mg/L. Although no species-specific clinical breakpoints or ECOFFs are currently available for *C. viswanathii*, these values exceed the CLSI epidemiological cut-off value reported for *C. tropicalis*, the closest available comparator species [41]. These data should not be interpreted as definitive evidence of clinical resistance, but they support the view that misclassification as *C. tropicalis* may lead to an inaccurate interpretation of the isolate’s biology, susceptibility profile, epidemiological significance and outbreak surveillance. Accurate species-level identification therefore remains essential for recognizing rare yeasts, detecting clusters, guiding antifungal susceptibility interpretation, and supporting infection-prevention measures [42].

### 4.5. Study Limitations and Practice Recommendations

This report has some important limitations to consider. Although we documented concordant *C. viswanathii* isolation from the patient and we used whole-genome sequenced *C. viswanathii* isolates as control strains [18], we did not perform WGS on our samples, which would have allowed a more definitive assessment of clonality. The number of clinical isolates was small, which reduces the robustness of inferences on susceptibility patterns. Beyond documenting a rare pathogen, this study suggests several priorities for practice and research. First, laboratories should implement explicit algorithms for panel-negative yeasts in high-risk settings, linking yeast-positive/BCID-negative blood cultures to extended identification by MALDI-TOF MS, implementing sequencing, and ensuring that MALDI libraries include well-curated spectra for uncommon *Candida* species. Second, FTIR and other rapid phenotypic fingerprinting tools merit systematic evaluation as adjuncts for species-level discrimination and outbreak first-line screening in medical mycology. Finally, multicenter collaborations are needed to better define the clinical spectrum, ecological reservoirs and resistance potential of *C. viswanathii* and related rare yeasts.

## 5. Conclusions

Our experience indicates that *C. viswanathii* is detectable with current technology, provided that MALDI-TOF databases and confirmation workflows are configured to recognize it. In this case, three MALDI systems and ITS sequencing all converged on the same identification at the species level.

FTIR analysis for the first time showed species genetically related as *C. viswanathii* and *C. tropicalis* are grouped into closely related clusters, albeit distinct from *C. parapsilosis* and *C. auris*.

In conclusion, based on our data, we can state that the identification of rare fungal infections depends less on the availability of sophisticated methods and more on the experience of high-level microbiology laboratory in setting robust algorithms linking gaps in fast microbiology to reliable identification and rapid outbreak surveillance.

## Figures and Tables

**Table 1:** MIC values of *C. viswanathii clinical* isolates. AB, amphotericin B; AND, anidulafungin; BAL: bronchoalveolar lavage; CAS, caspofungin; FLZ, fluconazole; ISA, isavuconazole; ITZ, itraconazole; MF, micafungin; POS, posaconazole. * First strain isolated from CVC blood culture. **^#^** *C. viswanathii* isolated from another center in Italy.

Sample ID	Sample Type	AND	MF	CAS	ISA	POS	VOR	ITZ	FLZ	AB
FPG 2277/25 *	Blood	0.06	0.03	0.12	0.12	0.12	0.12	0.25	2	0.5
FPG 2289/25	Blood	0.06	0.03	0.12	0.12	0.12	0.12	0.25	4	0.5
FPG 2296/25	Urine	0.03	≤0.008	0.06	0.06	0.25	0.12	0.12	4	0.25
FPG 2298/25	BAL	0.03	≤0.008	0.06	0.06	0.25	0.12	0.12	4	≤0.12
OPBG 1 ^#^	Blood	0.06	0.01	0.12	0.06	0.12	0.12	0.12	4	≤0.12
OPBG 2 ^#^	Blood	0.03	≤0.008	0.12	0.06	0.12	0.12	0.12	2	≤0.12
OPBG 3 ^#^	Blood	0.01	0.01	0.06	0.06	0.12	0.12	0.12	4	≤0.12

## Data Availability

The original contributions presented in this study are included in the article. All relevant information is presented in the case report. Further inquiries can be directed at the corresponding authors.

## Supplementary Materials

The following supporting information can be downloaded at: https://www.mdpi.com/article/10.3390/pathogens15070724/s1, Figure S1: Comparative chromogenic appearance of *Candida viswanathii* (left) and a reference *Candida tropicalis* ATCC 750 strain on CHROMagar™ Candida after 48 h of incubation at 37 °C. Both organisms produced thick, smooth, glistening blue-green/turquoise colonies, with minimal reproducible difference in chromogenic hue or colony morphology, indicating that in our setting the medium did not allow reliable macroscopic distinction between *C. viswanathii* and *C. tropicalis*; Figure S2: Spectral profiles of *Candida viswanathii* isolate FPG 2277. (a) Baseline-subtracted MALDI-TOF MS spectrum. (b) FTIR spectrum.
