# Supplementary material for: Unmasking Candida viswanathii in Panel-Negative Candidemia Through Integrated MALDI-TOF MS and FTIR Spectroscopy
Source: Pathogens. 2026 Jul 9;15(7):724. doi: 10.3390/pathogens15070724 (PMC13415169; doi:10.3390/pathogens15070724)
Supplement: Supplementary file 1 [file pathogens-15-00724-s001.zip › pathogens-4387053-supplementary.pdf]

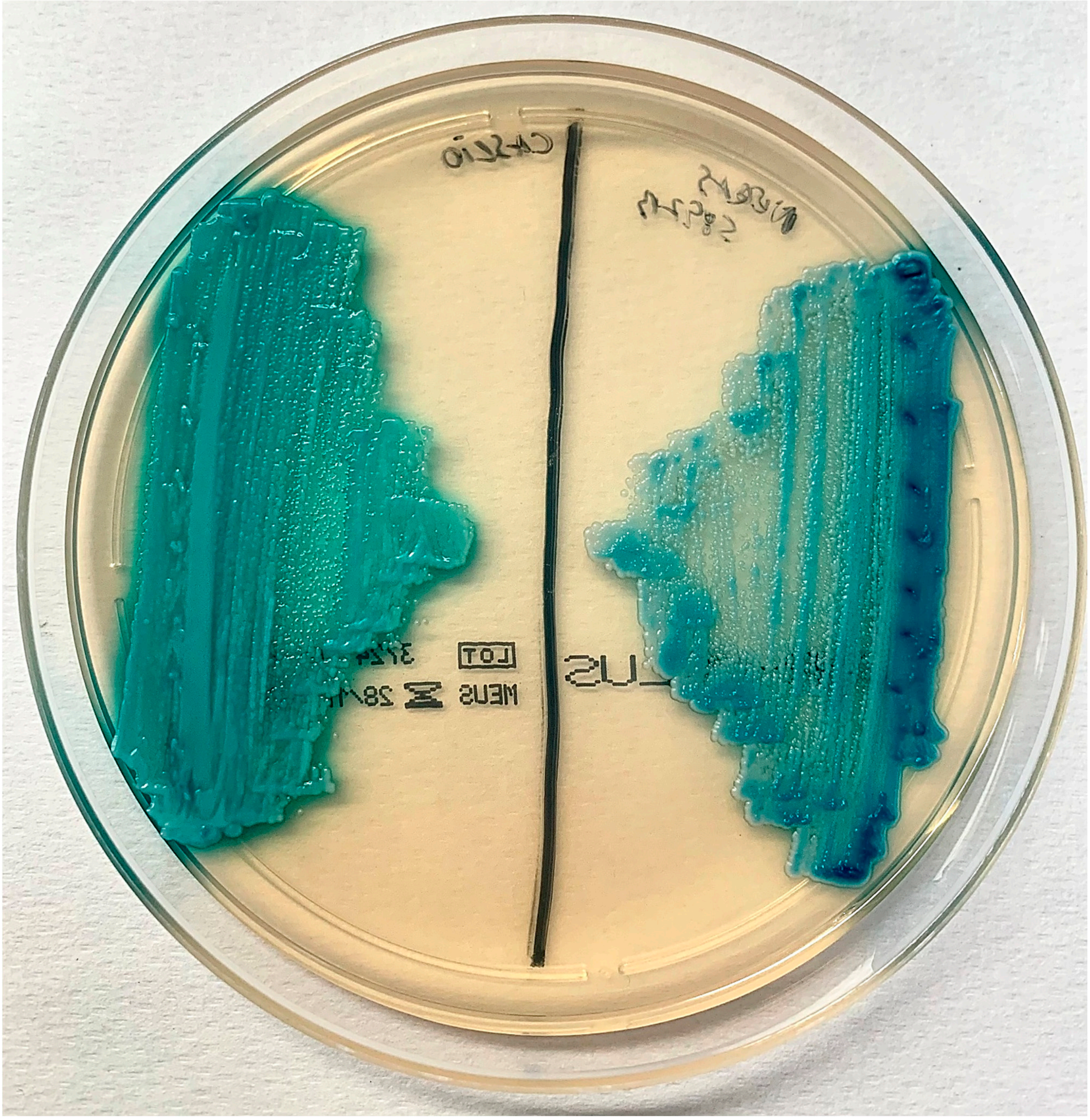

Figure S1. Comparative chromogenic appearance of *Candida viswanathii* (left) and a reference *Candida tropicalis* ATCC 750 strain on CHROMagar™ Candida after 48 h of incubation at 37 °C. Both organisms produced thick, smooth, glistening blue-green/turquoise colonies, with minimal reproducible difference in chromogenic hue or colony morphology, indicating that in our setting the medium did not allow reliable macroscopic distinction between *C. viswanathii* and *C. tropicalis*.

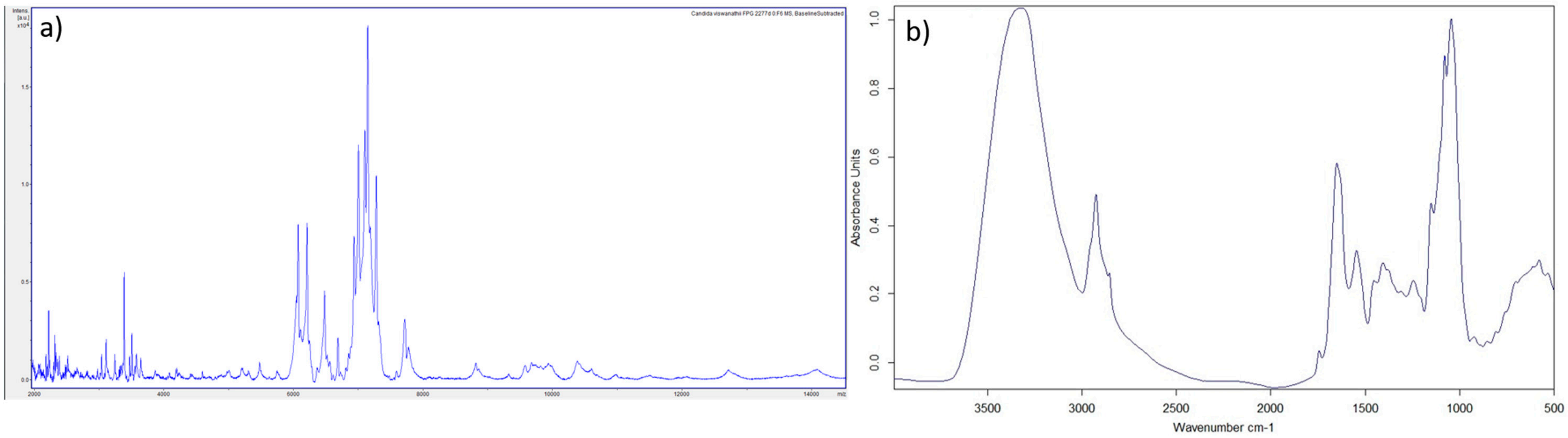

Figure S2. Spectral profiles of *Candida viswanathii* isolate FPG 2277. **a**) Baseline-subtracted MALDI-TOF MS spectrum **b**) FTIR spectrum
